# Unlocking the secrets of the invisible world: incredible deep optical imaging through in-silico clearing

**DOI:** 10.1038/s41377-023-01199-y

**Published:** 2023-06-27

**Authors:** Ting-Hui Xiao, Yuqi Zhou, Keisuke Goda

**Affiliations:** 1 Henan Key Laboratory of Diamond Optoelectronic Materials and Devices, School of Physics and Microelectronics, Zhengzhou University, Zhengzhou, 450052 China; 2 Institute of Quantum Materials and Physics, Henan Academy of Sciences, Zhengzhou, 450046 China; 3 Department of Chemistry, School of Science, The University of Tokyo, Tokyo, 113-0033 Japan; 4 Institute of Technological Sciences, Wuhan University, Wuhan, 430072 China; 5 Department of Bioengineering, University of California, Los Angeles, CA 90095 USA

**Keywords:** Imaging and sensing, Biophotonics

## Abstract

In-silico clearing enables deep optical imaging of biological samples by correcting image blur caused by scattering and aberration. This breakthrough method offers researchers unprecedented insights into three-dimensional biological systems, with enormous potential for advancing biology and medicine to better understand living organisms and human health.

Optical imaging is a powerful and widely used tool for studying biological and physiological phenomena in living specimens, thanks to its non-invasiveness and high resolving capability. However, its usefulness is limited when it comes to visualizing thick biological samples due to intrinsic limitations related to light-sample interaction processes, namely multiple scattering (MS) and sample-induced aberration (SIA)^1–3^. These processes generate optical noise that accumulates with light propagation, ultimately obscuring the effective optical signal for imaging. Addressing both MS and SIA is crucial to achieve high-resolution deep optical imaging.

Over the years, researchers have developed various approaches to tackle MS and SIA^1–5^. One method is to use gating operations, such as temporal or spatial gating, to suppress MS. For example, optical coherence tomography (OCT) utilizes the short temporal coherence time of its light source as a narrow temporal gating, while confocal microscopy utilizes the pinhole as a narrow spatial gating to suppress the contribution of MS^6,7^. However, gating performance is often degraded by SIA as it induces the loss of spatial coherence. Another approach is to use adaptive optics (AO) to correct SIA with dynamic correction elements, such as deformable mirrors and spatial light modulators^8,9^, but this method can be ineffective when strong MS is present. Therefore, there is a need for a method that can address both MS and SIA simultaneously.

In a newly published paper in Light: Science & Applications^10^, Osamu Yasuhiko and Kozo Takeuchi from Hamamatsu Photonics have proposed a novel method for achieving deep optical imaging, called in-silico clearing, which simultaneously addresses MS and SIA^10^. By identifying that both types of distortion stem from the same issue - heterogeneity in the distribution of refractive index (RI) - the authors developed computational algorithms to correct the image blur caused by MS and SIA, as shown in Fig. 1. The computational algorithms are highly efficient and enable efficient and high-resolution imaging over an extended imaging depth.

**Fig. 1:**
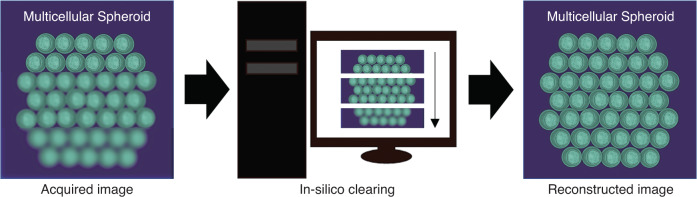
Schematic view of the in-silico clearing method

The computational algorithms consist of two main parts: partial reconstruction of the RI distribution and wave-backpropagation through the partial RI map. These parts are used to correct the image blur layer by layer. Initially, the algorithms use the measured complex optical field to reconstruct a clear RI distribution of the sample layer closest to the object lens and within the depth range where there is no image blur. Then, the algorithms numerically backpropagate the complex optical field, with removal of optical noise caused by MS and SIA in the current partial RI map, to reconstruct a clear RI distribution of the subsequent sample layer. This process is repeated until all sample layers are reconstructed, resulting in a clear RI tomography image of the thick biological sample.

The authors demonstrated the practical utility of the in-silico clearing method by using it to image a liver multicellular spheroid with a diameter of 140 µm. This method enables sharp resolution that can discriminate individual cells and resolve intracellular structures, such as nuclei and nucleoli, which are inaccessible with conventional methods. This resolving power enables the estimation of subcellular morphological changes inside the spheroid and the extraction of biologically relevant information. The authors have shown that lipid-droplet volume inside the liver multicellular spheroid can be quantitatively estimated, and the morphological changes inside the spheroid by treatment with an apoptosis inducer can be assessed. Furthermore, the method is general and applicable for various cell-type spheroids, including human lung cancer cell, human brain tumor cell, and mouse embryonal carcinoma cell spheroids.

The in-silico clearing method presents a comprehensive approach for deep optical imaging that can empower various optical imaging techniques, including bright-field imaging, quantitative phase imaging, and fluorescence imaging, with deep imaging capabilities. This offers non-invasive and high-resolution tools to study the architectures and functionalities of three-dimensional biological samples, such as three-dimensional cell culture models and native tissues^11^. The method holds potential for future in vivo imaging and the emergence of novel applications in the fields of biology and medicine^12^.
